# Digital Phenotyping in Bipolar Disorder: Which Integration with Clinical Endophenotypes and Biomarkers?

**DOI:** 10.3390/ijms21207684

**Published:** 2020-10-16

**Authors:** Laura Orsolini, Michele Fiorani, Umberto Volpe

**Affiliations:** Unit of Clinical Psychiatry, Department of Neurosciences/DIMSC, School of Medicine, Polytechnic University of Marche, 60126 Ancona, Italy; michele.fiorani88@gmail.com (M.F.); u.volpe@staff.univpm.it (U.V.)

**Keywords:** bipolar disorder, digital biomarkers, digital phenotyping, digital psychiatry, digital tool, phenotyping

## Abstract

Bipolar disorder (BD) is a complex neurobiological disorder characterized by a pathologic mood swing. Digital phenotyping, defined as the ‘moment-by-moment quantification of the individual-level human phenotype in its own environment’, represents a new approach aimed at measuring the human behavior and may theoretically enhance clinicians’ capability in early identification, diagnosis, and management of any mental health conditions, including BD. Moreover, a digital phenotyping approach may easily introduce and allow clinicians to perform a more personalized and patient-tailored diagnostic and therapeutic approach, in line with the framework of precision psychiatry. The aim of the present paper is to investigate the role of digital phenotyping in BD. Despite scarce literature published so far, extremely heterogeneous methodological strategies, and limitations, digital phenotyping may represent a grounding research and clinical field in BD, by owning the potentialities to quickly identify, diagnose, longitudinally monitor, and evaluating clinical response and remission to psychotropic drugs. Finally, digital phenotyping might potentially constitute a possible predictive marker for mood disorders.

## 1. Introduction

Bipolar disorder (BD) is a neurobiological disorder characterized by pathologic mood swings. Depressive episodes of BD are manifested through disturbed mood, psychomotor retardation, behavior change, decrease in energy levels, loss of interest in daily activities and sleep disorders. During a manic episode, patients may show an abnormal period of elevated or irritable mood, social interactions may be intense and/or the patient may become hyper-verbal [1]. Depending on the duration and severity of the episodes of mood elevation, the DSM-V classifies BD in type I and type II [1]. However, the profile of BD is more complex and heterogeneous, and includes mixed mood states, persistent mood instability, and cognitive dysfunction [2]. Overall, the lifetime prevalence of BD ranges from 1% to 2.4% [3]. A diagnosis of BD may determine a significant impact on subject’s overall disability, as the suicidality may occur in almost 4–19% of BD subjects [4] and the associated mortality, particularly due to a comorbid cardiovascular disease, may determine a significant increased risk of physical sequalae [5].

Digital phenotyping represents a new approach aimed at measuring the human behavior by using smartphones and personal device sensors, smartphone apps, keyboard interaction, and various features of subject’s voice and speech [6]. Data collected by a digital phenotyping smartphone application are divided into two categories: (a) active data (i.e., those usually collected by using a survey modality) which require an ‘active participation’ from the subject to be generated; and (b) passive data (for instance, those data collected by using global positioning system (GPS) traces), usually collected without any participation or action from the subject [7]. Digital phenotyping may theoretically enhance clinicians’ ability to early identify, diagnose, and manage any health conditions [8]. Moreover, a digital phenotyping approach may easily introduce and allow clinicians to perform a more personalized diagnostic and therapeutic approach to several physical as well as mental conditions [9]. Finally, it could be used as a tool for medical surveillance and redefine the disease expression in terms of ‘lived experiences’ of each ill individual [9]. The innovative and insightful approach applied by digital phenotyping appears to find an interesting and useful application in the field of psychiatry [10]. In fact, passive smartphone-based sensor data can be utilized to detect social anxiety symptom severity [11], to predict relapses in schizophrenia [12] and in depressive disorders [7], and to assess insomnia [9]. Moreover, smartphone-based real-time assessing can be used to identify distinct phenotypes of suicidal thinking [13]. In a similar way, digital phenotyping can be useful in BD, as we show in this paper. The digital phenotyping is in line with the new paradigm of the precision psychiatry, i.e., the new approach performed to help clinicians in customizing a psychiatric treatment for each patient, by integrating information about individual phenotypes and genotypes with biographical, clinical, and biological data [14]. A precision psychiatry approach would ideally allow clinicians to tailor clinical decision-making and stratify patients to each available treatment according to each one’s likelihood of treatment response and prognosis [15,16].

### Aims of the Paper

In BD, the trajectory of the illness is an essential element of the phenotype [17]. Digital phenotypes, if assessed by using smartphones or personal devices, may potentially offer an objective method of gathering data clinically useful to identify and describe individual phenotypes, by recording data and analyzing physiological patterns like speech [18], circadian rhythms [19], keyboard activity [20], daily self-assessment questionnaire [21], cognition speed, and affective responses [22]. The aim of the present paper is to investigate the role of digital phenotyping in BD. Therefore, we conducted a retrospective file review of all peer-review literature regarding the application of the digital phenotyping in BD. A review of all objectively selected, critically assessed, and synthetized evidence on available published data, available up to 30 August 2020, was undertaken.

## 2. Materials and Methods

### 2.1. Search Sources and Strategies

According to the recommendations of the Cochrane Collaboration [23], a systematic literature review was here conducted and documented in accordance with the Preferred Reporting Items for Systematic Reviews and Meta-Analyses (PRISMA) guidelines [24]. MEDLINE, PubMed, Cochrane Library, and Scopus online databases (last update: 30 August 2020) have been consulted by combining the following free text terms and exploded MESH headings for the topics of: “*bipolar disorder*”, “*digital phenotyping*” as follows: (*digital phenotyping* [Title/Abstract]) and (*Bipolar Disorder* [Title/Abstract]) OR (*Mood Disorder* [Title/Abstract]). The strategy was first developed in MEDLINE and then adapted for use in the other databases, without any language restriction or on the year of publication applied. Further studies were retrieved through hand-searches of reference listings of relevant articles and consultation with experts in the field.

### 2.2. Study Selection

All studies were evaluated specifically investigating BD, and by excluding all articles addressing other mood disorders, including depression (unipolar) and major depressive disorder (MDD). Titles and abstracts were examined. Consequently, we acquired full texts of potentially relevant papers. Two reviewers (LO and MF), working independently and in parallel, examined the papers and judged if they met inclusion criteria. We excluded duplicate publications. All experimental and observational study designs were screened, including case reports. Randomized, controlled clinical trials involving humans were prioritized, whilst preclinical/animal studies were excluded. We excluded all narrative and systematic reviews, letters to the editor, and book chapters.

### 2.3. Data Extraction and Management

After selection of the relevant studies, LO and MF independently extracted the data on participant characteristics, intervention details and outcomes measures. Disagreements were resolved by discussing the content of the paper with a third member of the team (UV). Finally, data were systematized using an Excel spreadsheet developed specifically for this study.

### 2.4. Characteristics of Included Studies

**Figure 1:**
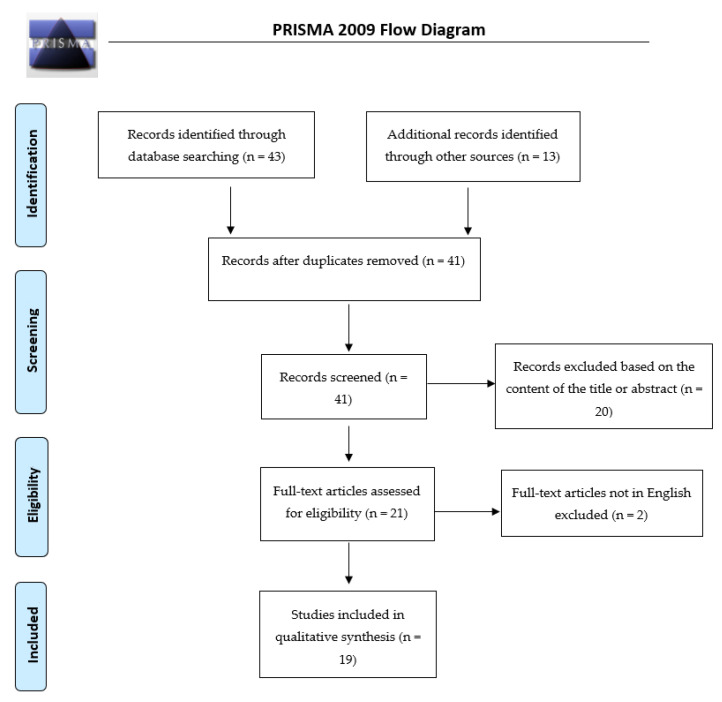
PRISMA 2009 flow diagram.

From 56 potentially relevant records, 15 studies were eliminated due to duplication. The remaining 41 underwent more scrupulous check. Subsequently, 20 were excluded based on the content of the title or abstract, and 2 because they were in languages other than English. Overall, 19 studies met the inclusion criteria (see Figure 1). The main characteristics of the studies are summarized in Table 1.

## 3. Results

To date, there are few studies about digital phenotyping in BD, and most of them mainly focused on how digital phenotyping may predict mood change. Literature so far published on the topic refers to a really limited timeframe ranging from April 2013 to 2020, by suggesting that the research in digital phenotyping, particularly in the field of BD, is still at its earliest stages. Overall, literature here retrieved may be ideally classified, according to the type of data collection and processing, alongside six research key-points, as follows: (a) studies investigating the development of mood prediction algorithms by using a digital phenotyping approach [19,21]; (b) studies investigating the association between mobile phone keyboard metadata and mood disorders [20,25]; (c) studies evaluating the relationship between specific patterns of speech features and mood disturbances [18,26,27,28]; (d) studies investigating the correlation (if any) between automatically generated objective smartphone data and the mood [22,29,30,31,32]; (e) studies investigating the development of a healthcare app for BD [33,34,35,36,37] and all further future applications of the digital phenotyping in the treatment of BD [38]. The main findings of each paper here retrieved are summarized in Table 1.

### 3.1. Studies Investigating the Development of Mood Prediction Algorithms by Using a Digital Phenotyping Approach

Cho et al. [19] described a mood prediction algorithm by using a machine learning tool by processing passive digital log data (collected via smartphones and wearable devices) about individual activity, sleep, the intensity of light exposure, and the heart rate and compared them with self-recording daily mood scores and conventional clinical assessments, in a sample of patients with mood disorders (*n* = 55), including BD-type I (*n* = 18) and BD-type II (*n* = 19), according to the DSM-5 criteria [1]. The study is part of the Mood Disorder Cohort Research Consortium (MDCRC) study, a multicenter prospective observational cohort study investigating early-onset mood disorders in Korea [39]. Patients were assessed by using standard clinical assessments and a specifically developed eMoodchart smartphone app. Findings confirmed the utility for patients with BD to manage their activity levels and exposure to light to coordinate with their circadian rhythm to maintain a stable mood state and that the variations related to circadian rhythms can meaningfully reflect the mood state of the subject. The overall prediction accuracy for the mood state was relatively good, particularly for BD-type II (82.6%, 74.4%, and 87.5% of accuracy for with 0.919, 0.868, and 0.949 area under the curve for no episode, depressive episode and hypomanic episode respectively).

Busk et al. [21] examined the feasibility of a smartphone-based system in mood forecasting, i.e., predicting the mood one or more days ahead based on historical data collected through daily mood scores digitally collected from a sample of 84 BD patients, recruited during the MONARCA II randomized clinical trial [40]. Patients were randomized to either using a smartphone-based monitoring system (the Monsenso system) for daily self-monitoring (the intervention group) or treatment as usual (the control group). Busk et al. [21], by using a hierarchical Bayesian regression model and a multi-task learning method, digitally recorded any individual differences of each participant as well as forecasted their respective mood in a period up to seven days. Their findings indicated that the subjective mood might represent a valid indicator of the mental state in BD patients, by potentially becoming a clinically relevant feature applicable for daily monitoring and forecasting in BD.

### 3.2. Studies Investigating the Association between Mobile Phone Keyboard Metadata and Mood Disorders

Further studies attempted to analyze passively collected mobile phone keyboard metadata with the aim to predict manic and/or depressive signs and/or symptoms in BD patients [20,25].

A pilot study carried out by Cao et al. [25] explored the possible connections between BD and mobile phone usage loaded with a custom keyboard able to collect metadata consisting of keypress entry time and accelerometer movement, by investigating whether these metadata could be used to predict the presence and severity of a mood disturbance. Data were collected from the BiAffect study [20], by recruiting 40 subjects (7 BD-type I, 5 BD-type II, 8 BD not otherwise specified, and 20 healthy controls) for a period of 8 weeks. The authors proposed an end-to-end deep architecture, named DeepMood, to model mobile phone typing dynamics, by demonstrating that an extremely accurate measure of depression can be achieved in less than one minute using level mobile phone typing dynamics.

In a prospective 8-week cohort study, Zulueta et al. [20] investigated the relationship between mobile phone keyboard activity and mood disturbances in 19 subjects with bipolar spectrum disorder [41], by using a mobile phone loaded with a customized keyboard (named ‘BiAffect’) that passively collected keystroke metadata, time, and accelerometer displacement. Subjects were enrolled from the Prechter Longitudinal Study of Bipolar Disorder, a naturalistic longitudinal study [42], by specifically including only subjects with a current Android mobile phone, asserting familiarity with the Android operating system, with sufficient vision and no impaired fine motor abilities. The findings supported the hypothesis that each mood state (i.e., depression, manic, mixed) in BD, was positively correlated with a specific pattern of changes in the mobile phone usage (e.g., increased accelerometer activity, overall number of sessions, the rate of typing errors, diurnal vs. nocturnal pattern of phone activity, etc.).

### 3.3. Studies Evaluating the Relationship between Specific Patterns of Speech Features and Mood Disturbances

Muaremi et al. [28] explored the feasibility of voice analysis during phone conversations, to discriminate and predict a BD episode, by using a unique combination of phone call statistics (i.e., number of phone calls/day, sum of the duration of all phone calls/day, average duration of the phone calls, standard deviation of phone call durations, minimum/maximum duration of all daily phone calls, % of phone calls happening in the morning/night), social signals (e.g., average speaking length, average number of speaker turns, average speaking turn duration, average number of short turs/utterances, % of speaking from the total conversation, short turns/utterances per length in minutes) and acoustic emotion properties (e.g., root mean square frame energy, mel-frequency cepstral coefficients, pitch frequency F_0_, harmonics-to-noise ratio and zero-crossing-rate) with a subjective self-assessment, by recruiting 12 BD subjects. The authors revealed that from the three categories, acoustic features showed best performance in terms of state recognition followed by the social cues. In particular, the speaking length and phone call length, the harmonics-to-noise ratio value, the number of short turns/utterances, and the pitch frequency F_0_ represented the most clinically relevant variables for predicting mood states in BD subjects.

McInnis et al. [18] explored the role of digital phenotypes in patients with rapid cycling BD, after an analysis of speech segmentation, including silences, captured by using two smartphone models pre-loaded with ‘PRIORI’, an app that recorded and encrypted all outgoing speech from all phone calls devices. The authors demonstrated a good predictability in identifying each mood state in BD subjects.

Karam et al. [27], through a pilot analysis, described methodology to collect unstructured speech continuously and unobtrusively via the recording of day-to-day cellular phone conversations of 6 BD-type I patients with a history of rapid cycling, recruited from the Prechter Longitudinal study of Bipolar Disorder at the University of Michigan [43] and followed-up until 6 months to a year. Each participant was provided with a smartphone with an unlimited call/data plan for personal use and encouraged to use the phone as their primary mode of contact. The phone was pre-loaded with an application able to record only participant’s outgoing speech conversations, whenever they make or receive a phone call. Participants’ mood state was obtained using weekly phone-based interactions with a clinician administering clinician rating scales. The findings demonstrated how using a speech-based classifier could significantly differentiate a hypomanic and/or depressive episode by a euthymic phase.

Gideon et al. [26], within the PRIORI Dataset aimed at collecting smartphone conversational data, recruited 37 subjects affected with rapid-cycling BD, BD-type I and BD-type II from the Prechter Longitudinal study of Bipolar Disorder at the University of Michigan [43]. Participants were enrolled for 6 to 12 months and were provided with an Android smartphone with the secure recording application PRIORI app installed. A clinician administering standard rating scales weekly-assessed participants. Gideon et al. [26] described methodologies for use during preprocessing, feature extraction and data modeling to better perform a study able to statistically significantly provide higher performance, without reporting any findings.

### 3.4. Studies Investigating the Correlations between Automatically Generated Objective Smartphone Data and Mood

Grunerbl et al. [31] developed a smartphone sensor-based app that automatically record all BD-relevant data in the phone background without requiring any input by the user. The app is able to extract several background data (e.g., phone call features, speech and voice features, GPS data, acceleration, movement features, etc.), then combined to produce a final classification for each daily usage. The system is able to detect early changes in the state of a BD patient, by not requiring long periods of training and calibration by patients.

A pilot study by Abdullah et al. [29] evaluated the achievability of an automatically assessment of the social rhythm metric (SRM), i.e., a clinically validated marker of stability amongst individuals with a mood disorder, by using a customized smartphone app which passively collected behavioral (e.g., speech, activity, SMS, and call log) and contextual data (e.g., location). Based on the collected data, the authors employed machine-learning techniques to model and predicted markers of rhythmicity in the daily life of BD patients. Participants were provided with MoodRhythm, a tool able to track both subjective rating and automatically sensed data, to track their social rhythms. Findings reported that location, distance traveled, conversation frequency, and non-stationary duration can be used to infer the SRM score.

In a prospective community study, Palmius et al. [32] tried to identify the periods of depression in a sample of individuals affected with BD (*n* = 22) versus healthy controls (*n* = 14) observed over 3 months, by using the geolocation movements recorded from each individual mobile phone. Participants were provided with an Android-based smartphone with a custom app installed that records the anonymized geographic location of the smartphone. Location data were recorded by using different location data sources including GPS, nearby wireless network access points, and triangulation of the distance to any nearby cellphone towers. Passively recorded objective location data were pre-processed by detecting and removing imprecise data and compared with weekly self-assessments questionnaire on depressive symptomatology, by using QIDS-SR_16_ (self-report 16-item quick inventory of depressive symptomatology) [44]. The findings demonstrated that it is possible to detect depressive episodes in BD subjects with 85% accuracy by using geographic location recordings alone. Geographic location movements can provide a useful metric for the identification of depressive symptoms in BD subjects in community settings, according to the study by Palmius et al. [32].

A pilot study by Beiwinkel et al. [30] used a smartphone-based monitoring system, named Social Information Monitoring for Patients with Bipolar Affective Disorder (SIMBA), to collect both objective passive sensor data (i.e., GPS for the distance traveled per day, cell tower movement as an indicator of location changes, and accelerometer to measure the users’ device activity) and subjective self-report data (by administering clinical rating scales to assess clinical depressive and manic symptoms). Analyzing the correlations between smartphone-based data, collected in a framerate of 12 months, and externally clinically assessed manic and depressive symptoms, the findings described an association between objective and subjective measurement, even though not all smartphone-based measures predicted the occurrence of BD symptoms above clinical threshold. In particular, lower self-reported smartphone-based mood variables could foresee overall levels of clinical depressive symptoms (*p* < 0.001). A decreased activity of social communication (e.g., outgoing text messages) and the decline in physical activity (measured by cell tower movements) could predict an increase in clinical depressive symptomatology as well (respectively, *p* < 0.001 and *p* = 0.03). Lower physical activity (e.g., distance travelled) and higher social communication registered on the smartphone could predict levels of clinical manic symptoms (respectively, *p* = 0.03 and *p* < 0.001). An increase in clinical manic symptoms was predicted by a decrease in physical activity on the smartphone (*p* < 0.001).

Dargél et al. [22] proposed a research protocol for an open label, nonrandomized trial enrolling 93 subjects with BD type I/II according to the DSM-5 criteria [1] at different phases (31 depressive, 31 euthymic, and 31 hypomanic), aiming at exploring the correlations between clinically assessed manic/depressive symptoms and smartphone-based behavioral variables (by using a specifically designed smartphone app, named Toi Même). The Toi Même app combines passively collected behavior data (e.g., daily motor activities such numbers of steps for day, distance travelled, etc.); ecological momentary assessments through two gamified tests to evaluate cognition speed (Quickbrain) and affective responses (Playimotions) in real life contexts; and a weekly assessment (including validated self-rating scales).

### 3.5. Studies Investigating the Development of Healthcare Apps for BD

In 2013, Bardram and Faurholt-Jepsen et al. [33] developed the MONARCA system—a personal monitoring system for BD patients. This smartphone application collects subjective self-reported data and objective sensor data. The self-assessment items included mood, sleep, subjective activity, medicine adherence, universal and early warning signs, use of alcohol, and stress. The objective data are collected via sensors in the phone and includes physical activity data as measured by the accelerometer and social activity as measured by the number of in and outgoing phone calls and text messages. In addition, the app provides a feedback system that can be visualized through simple graphs. In a 14-week clinical trial involving 12 adult participants affected with BD, the authors found that the patient’s adherence to the digital self-assessment improved compared to using paper-based forms, the MONARCA system was overly considered very easy to use, by reaching a very high-perceived usefulness by patients. Since the development of the MONARCA system by Bardram and Faurholt-Jepsen et al. [33], further studies were performed by using the MONARCA system as a research tool [34,35,36,37].

Alvarez-Lozano et al. [34] in a clinical trial recruiting 18 BD subjects, investigated app usage patterns within the smartphone and their correlation with the episode of BD patients. Patients were provided with a smartphone containing MONARCA system and were monitored over a period of 5 months. The MONARCA system collected data while running on the background, sampling accelerometer sensor, WiFi card, running applications, screen status, Bluetooth and microphone in a privacy-preserving manner. The authors found a strong correlation of patterns of app usage with different aspects of patient’s self-reported state like the mood, sleep, and level of irritability.

In the first randomized controlled single-blinded trial that examine the effect of electronic self-monitoring in patients with BD, Faurholt-Jepsen [35] reported that the MONARCA system may be an effective tool able to early recognize warning signs and symptoms of a hypomanic/manic episode in BD subjects, even though it was less effective in identifying early warning signs and symptoms of a depressive episode in BD subjects.

In a prospective cohort study, which used the smartphone software MONARCA, Faurholt-Jepsen [36] investigated whether objective smartphone-based data could distinguish between patients with BD and healthy controls (HC), in order to potentially identify if this tool may represent a valid diagnostic digital marker in discriminating a BD. They hypothesized that automatically generated objective smartphone data would be able to discriminate: (1) between patients with BD during euthymia compared with HC; (2) between patients with BD during depressive state and manic or mixed state compared with HC; and, (3) between patients with BD overall compared with HC. The results showed how objective smartphone data (i.e., the number of text messages/day, the duration of phone calls/day) increased in patients with BD (during the euthymia, depressive and manic or mixed state, and overall) compared with HC individuals. Therefore, they concluded that objective smartphone-based variables would represent a diagnostic behavioral marker in BD.

In a second randomized controlled single-blinded trial, Faurholt-Jepsen et al. [37] examined the effect of a smartphone-based system on the severity of depressive and manic symptoms in BD patients. The results showed that smartphone-based monitoring and real-time mood prediction did not reduce the severity of symptoms in BD. Nevertheless, in patient-reported outcomes, patients in the intervention group (using the MONARCA system) described an improved quality of life and a reduced perceived stress, compared to healthy controls. Instead, participants in the intervention group had higher risk of depressive episodes, but a reduced risk of manic episodes.

### 3.6. Studies Investigating All Further Future Applications of the Digital Phenotyping in the Treatment of BD

Amongst the literature so far published and retrieved, in only one study did the authors investigate the therapeutic potentiality of digital phenotyping in BD by discussing a large pan-European multidisciplinary collaborative research protocol named R-LiNK study [38]. In particular, by combining systematic clinical syndrome subtyping with the examination of multi-modal biomarkers (or biosignatures)—including omics, neuroimaging, and actigraphy—the authors investigated if a digital phenotyping approach may be useful in the early prediction of lithium response, non-response, and tolerability amongst BD patients. In detail, the combination of daily self-ratings (e.g., mood, energy, and activity), continuous actigraphic recordings of sleep–wake cycles and a prototype device that allows home-based measurement of salivary lithium levels, may help clinicians to refine the individual phenotype of clinical response and, hence, potentially translate this finding into a personalization of lithium treatment.

## 4. Discussion

### 4.1. Key Findings and Comparison with the Literature

In the last two decades, the rapid expansion of digital technology, particularly the ubiquity of smartphones reaching up to 77% of US population out of around 95% of US population who reported owning a mobile phone, could make them a potential real-time digital platform for the diagnosis, monitoring, and relapse detention of psychiatric diseases, including BD [2,8,45,46]. In fact, there is a plethora of data that is generated and recorded during the interaction between an individual and his/her smartphone or personal device, some data that might occur with minimal participation (active) whilst other data with little or no burden for them (passive) [8,47]. The collection and analysis of passive and continuous behavioral information generated from mobile devices and smartphones allows a context-based, ecologically valid profiling of the digital phenotype construct of everyone [9]. Digital phenotyping is defined as the ‘moment-by-moment quantification of the individual-level human phenotype in its own environment’ by using mobile devices or apps from smartphones [8,12]. During the last decades, the research in mood disorders has gradually shifted to more quantifiable variables (i.e., epigenetic, genetic, molecular, and structural imaging characterization) rather than behavioral aspects [6,8]. Therefore, the complementary approach of digital phenotyping may provide a solid strategy to better identify and stratify mood disorders, including BD, and improve outcome prediction, including treatment response, relapse, and recurrence [8,48].

Overall, literature so far published and here retrieved pointed out on a set of potential applications of the digital phenotyping in the field of mood disorders, particularly in BD. Firstly, digital phenotyping may be used as a diagnostic predictive marker in BD, by identifying BD specific traits for a rapid and accurate diagnosis. Secondly, digital phenotyping may be considered as a tool to predict longitudinal outcomes, by considering how BD subjects may oscillate in mood, energy levels, and cognition within hours, days, and months [49]. In this context, digital phenotyping could significantly improve the early identification and intervention in potentially life-threatening conditions, including fluctuations in suicidal ideation and thoughts of death [50], the dimension of the affective instability comprising emotional intensity, emotional lability, and ability to control shifts in mood [8,49]. Moreover, it may be a valid tool for clinical characterization, course of illness (i.e., detection of subgroups of BD patients), prediction of critical outcomes in illness course (i.e., relapse, recurrence, resilience), to early detecting, monitoring and predicting treatment response, non-response, remission, and treatment tolerance (i.e., identification of predictors of side effects) as well as a prediction tool for the identification of high-risk BD subjects [8,19,21,45]. At this regard, it may help clinicians as well in refining the clinical response phenotype and could translate into the personalization of lithium treatment [38].

The development of a mood prediction algorithm, by using either a daily clinical self-assessment collected via smartphone [21] or a passive digital log data [19] can be used to identify early warning signs and/or symptoms during a clinical treatment of a BD. Furthermore, accurate symptom forecasting could be extended to detect the risk of relapse of a major affective episode in BD subjects [19]. Despite exploring the development of a mood prediction algorithm in the field of BD could be extremely useful for clinicians for both early identifying a relapse occurrence and monitoring the clinical response to a treatment; to date, only two studies have been published with a limited sample size and one study recruited a mixed sample including other mood disorders as well [19,21].

Another application of digital phenotyping in BD is the development of smartphone-based healthcare apps designed for performing a clinical self-assessment that can help patients in estimating and monitoring their own symptoms and receiving a feedback about their mental health [33,34,35,36,37,38]. For instance, the MONARCA System [33] evaluated different BD-related clinical variables, including the mood, sleep, subject’s activity and therapy adherence. However, despite the MONARCA system was also implemented in further studies and overly considered easy to use by BD patients, there is still lacking scientific evidence in the generalizability and applicability of the system in the real-life [35,36,37].

An additional digital phenotyping approach developed in the field of BD, is represented by an objective monitoring of symptoms of BD, by recording passive data collected by using specific smartphone sensors used to infer patient’s mood state and behavior [18,20,25,27,28]. For instance, Mauremi et al. [28] and McInnis et al. [18] investigated how processing and analyzing voice features during a call can theoretically predict a mood state in BD subjects whilst Karam et al. [27] suggested how these passive data may differentiate a hypomanic, depressive, or euthymic state. Moreover, smartphone-based keyboard interaction dynamics appear to be modified depending on the mood state of the subject [20,25]. Social activity patterns (e.g., distance traveled, frequency of conversation) can be used to infer rhythmicity, a key marker of wellbeing for individuals with BD [29]. Geolocation movements recorded via GPS can detects depressive episode [32]. Finally, smartphone-based recording of subject’s physical activity may represent a warning sign for phase transitions in BD subjects [30]. Overall, the advantage of this approach is that it does not require any input/feedback from the patient, which is often unreliable and carries the risk of being biased [31]. However, this digital phenotyping approach may arise some concerns regarding the use of a passive monitoring related to stigma and privacy [51]. Despite the possibility of digital phenotyping approach being promising—becoming a potential diagnostic behavioral and mood digital predictive marker able to theoretically supplement, assist, and facilitate the rapid assessment and diagnosis of BD subjects—literature so far published displays several methodological challenges which may greatly limit the generalizability of these findings, including the lack of consistent sample size, mixed clinical features of the sample, and missing evidence in real-life clinical settings.

### 4.2. Main Strengths and Limitations

Despite its originality and illuminating poorly investigated topic, there are several limitations with this study that should be drawn up. Firstly, despite the intent to perform a systematic review, the limited numbers of studies published and here retrieved together with an extremely heterogeneous methodological variability do not allow a systematic approach in collecting and analyzing findings here retrieved. In fact, the topic of the digital phenotyping in the field of BD is poorly investigated and extremely variegated in the application; hence limiting the possibility to critically compare all variables investigated by each study here included. Furthermore, most studies include a very small sample size, assessment tools used that are not homogeneous and clinically mixed samples (i.e., some studies include also other mood disorders). There are few randomized clinical trials. Furthermore, three studies here discussed are still ongoing or they discuss only methodology/research protocol [22,26,38]. Moreover, studies do not include measures related to the improvement of the feasibility of apps neither some reflection about clinical measures to be implemented regarding the potential detrimental long-term impact in using a digital phenotyping approach on the mood, levels of energy, level of functioning, overall clinical course, treatment response/remission, patient’s compliance, and adherence. Moreover, studies do not evaluate the potential impact that using a digital phenotyping approach may have on the occurrence of a comorbid web/app-based psychopathology. Furthermore, studies do not investigate potential legal issues related to app or technology errors, and technical issues related to privacy and security regarding the collection of passive smartphone data. In addition, issues related to radiofrequency microwave radiation exposure are omitted, including cellphone safety limits and emissions when cellphones touch the body. Moreover, potential negative effects of smartphone-based monitoring and mood prediction should be investigated. Finally, studies do not specifically address the feasibility of digital phenotyping in assessment, diagnosis, monitoring, and treatment of older BD patients as well as in children and/or adolescents.

### 4.3. Relevance of the Findings and Implications for Practice and Research

In the light of the recent COVID-19 outbreak which placed all clinicians in a need to perform alternative (digital) modalities to ensure a continuous monitoring and follow-up of psychiatric patients, the present review and field of research, being innovative and still under investigated, represents an insightful perspective, worth being further deepened and investigated. These findings not only serve to investigate the current research situation in terms of literature published regarding the clinical applicability of digital phenotyping approaches in the field of BD, but also to underline how still inconsistent and poor the research is in this field of digital psychiatry. These findings may assist the decision-makers to implement research strategies and more homogeneous methodologically studies. Further studies are needed to better evaluate the efficacy and external validity of smartphones and wearable devices in extrapolating digital biomarkers for mood disorders, e.g., in discriminating different phases of a BD (mixed, manic, depressive) and in providing predictions for clinically relevant outcomes (i.e., clinical course, recurrence/relapse and therapeutic response/remission to specific drug). Moreover, future studies may be performed in order to integrate the digital phenotyping approach into the Research Domain Criteria (RDoC) paradigm proposed by the National Institute of Mental Health (NIMH) [6,52,53,54]. At this regards, digital phenotyping may offer real-time smartphone-based clinical self-assessment, behavioral, and physiological units of analysis useful for an RDoC analysis [54]. Particularly, digital phenotyping may be useful in deepening the RDoC-based negative valence systems and social processes domains [54].

Furthermore, several ethical concerns should be carefully evaluated. Firstly, most apps fail to conform to clinical guidelines, hence, being potentially harmful for the patient who could not adequately receive an effective treatment intervention or a delayed proper treatment [55]. Secondly, one could argue that there is the need to preventively evaluate a patient’s ability to preserve autonomy, to ensure privacy and confidentiality, to achieve digital informed consent (or to withdraw consent), including a full understanding of the scope of digital phenotyping and security systems for protected and safe storage of the recorded patient’s data. Lastly, clinicians should consider affordability and ensure equitable access to care, considering the costs associated with the use of mobile devices and/or smartphones [56].

## 5. Conclusions

Digital phenotyping as a complement to traditional psychiatric practices (i.e., clinical-based interviews, mental status examination, psycho-diagnostic assessment) could potentially improve the diagnosis (early identification of a newly diagnosed mood disorder and early identification of the occurrence of a manic/hypomanic/depressive/mixed episode within a BD), clinical course, detection of a relapse/recurrent episode, prognosis, and treatment response/remission of a BD. Moreover, it could contribute to a better characterization of treatment response and to make a more personalized therapy, in line with the current paradigm of the precision psychiatry. By monitoring patients in a passive and a real-time mode during a long-term follow-up period, smartphones may have the potential to ‘capture’ dynamic patterns of behaviors and mood states that can alert the patients themselves, their caregivers, and their mental healthcare providers. Such predictive identification could enable timely and effective interventions in the field of BD. Digital phenotyping is still in a blossoming state, and to date there is still a dearth of scientific literature regarding the applicability of digital technologies in the field of mental health disorders. Therefore, further studies should be performed to better understand how digital phenotyping could become a standard clinician tool in psychiatry.

## Figures and Tables

**Table 1:** Summary of studies.

Reference	Study Design	Sample Features	Diagnostic Criteria	Objectives	Methodology	Main Findings
[15]	Prospective cohort study	60 pts	BD-rapid cycling (*n =* 51) HC (*n =* 9)	To monitor moods over extended periods of time using speech.	Length of study: unspecified Intervention group: 60 pts Type of app: PRIORI Type of recorded data: passive smartphone voice and speech data	Digital phenotypes derived from speech captured from mobile devices predict mood states.
[16]	Prospective observational cohort study	55 pts	MDD (*n =* 18) BD-I (*n =* 18) BD-II (*n =* 19) (DSM-5)	To investigate a mood prediction algorithm developed with machine learning using passive data phenotypes based on circadian rhythms.	Length of study: 2 years Intervention group: 55 pts Type of app: eMoodchart Type of recorded data: (a) Subjective daily self-report mood data (b) Passive digital log data (activity, sleep, heart rate, light exposure)	The utility for patients with BD to manage their activity levels and exposure to light to coordinate with their circadian rhythm to maintain a stable mood state. The variations related to circadian rhythms can meaningfully reflect the mood state of the subject.
[17]	Prospective cohort study	9 pts (8 F, 1 M)	BD-I (*n =* 5) BD-II (*n =* 4) (DSM-IV-TR)	To investigate the relationship between mobile phone keyboard activity and mood disturbance in subjects with BD.	Length of study: 8 weeks Intervention group: 9 participants Type of app: BiAffects Type of recorded data: passive smartphone data (keystroke metadata, time and accelerometer displacement)	Mood states in bipolar disorder appear to correlate with specific changes in mobile phone usage.
[18]	RCT	84 pts (52F, 32M), 21–71yy	BD (unspecified type) (ICD-10)	To examine the feasibility of forecasting daily subjective mood scores based on daily self-assessments collected from BD patients via a smartphone-based app.	Length of study: 9 months Intervention group: 84 pts Type of app: MONARCA Type of recorded data: daily smartphone-based self-monitoring data	Subjective mood may represent a valid indicator of the mental state in BD patients.
[19]	nonrandomized trial	93 pts	BD-I BD-II (DSM-5)	To investigate the correlations between clinically rated mood symptoms and mood/behavioral data automatically collected using a specifically designed smartphone app.	Length of study: 3 months Intervention group: 93 pts Type of app: Toi Même Type of recorded data: (a) Subjective weekly self-report mood data (b) ecological momentary assessments (measured by 2 gamified tests) (c) Passive smartphone data: motor activity (measured by motion sensor)	Ongoing Study. Completion of the study is estimated in December, 2021.
[22]	Pilot study, 8-weeks prospective	40 pts	BD-I (*n =* 7) BD-II (*n =* 5) BD-NOS (*n =* 8) HC (*n =* 20) (DSM-IV-TR)	To explore the possible connections between BD and mobile phone usage.	Length of study: 8 weeks Intervention group: 40 pts Type of app: DeepMood Type of recorded data: passive smartphone’s keyboard metadata (keypress entry time and accelerometer movement)	An extremely accurate measure of depression can be achieved in less than one minute using level mobile phone typing dynamics.
[23]	Prospective cohort study	37 pts	BD-rapid-cycling BD-I BD-II	To explore speech collected from phone recordings for analysis of mood in individuals with BD.	Length of study: 6–12 months Intervention group: 37 pts Type of app: PRIORI Type of recorded data: passive smartphone voice and speech data	Authors develop a methodology for use during preprocessing, feature extraction and data modeling to better perform a study able to statistically significantly provides higher performance.
[24]	Pilot study	6 pts	BD-I with a history of rapid cycling (i.e., characterized by 4 or more episodes per year of mania, hypomania, or depression)	To determinate the feasibility of detecting the mood state assessed during the evaluation call using recording cellular phone conversations.	Length of study: 6–12 months Intervention group: 6 pts Type of app: app that records only the participant’s outgoing speech (unspecified name) Type of recorded data: passive smartphone voice and speech data	A speech-based classifier can significantly differentiate a hypomanic and/or depressive episode by a euthymic phase.
[25]	Pilot study	12 pts, 18–65yy	BD (unspecified type)	To discriminate and predict a BD episode, by using voice analysis during phone conversations.	Length of study: 12 weeks Intervention group: 12 participants Type of app: unspecified Type of recorded data: (a) daily subjective self-assessment (b) passive smartphone data (phone call statistics, social signals and acoustic emotion properties	Speaking length and phone call length, the harmonics-to-noise ratio value, the number of short turns/utterances and the pitch frequency F0 represented the most clinically relevant variables for predicting mood states in BD subjects.
[26]	Pilot study, 4-week prospective	9 enrolled pts (5 F, 4 M), of which 7 included pts (5 F, 2 M), 24–65 yy	BD-I (*n =* 1) BD-II (*n =* 5) BD-NOS (*n =* 1)	Evaluate the feasibility of automatically assessment of SRM for BD patients by using passively-sensed data from smartphones.	Length of study: 4 weeks. Intervention group: 7 pts Type of app: MoodRhythm Type of recorded data: (a) subjective self-report (b) passive smartphone sensors data (accelerometer, microphone, location, communication information)	Location, distance traveled, conversation frequency and non-stationary duration can be used to infer the SRM score.
[27]	Pilot study, 12-months prospective	32 enrolled pts, of which 12 included pts	BD-I or BD-II (DSM-IV-TR), at least 18 years of age, sufficient knowledge of the German language, and basic competence in using mobile devices	To investigate whether smartphone measurements predicted clinical symptoms levels and clinical symptom change in BD.	Length of study: 12 months Intervention group: 12 pts Type of app: SIMBA Type of recorded data: (a) Subjective daily self-report mood data (b) Passive smartphone data: physical activity (measured by the accelerometer, GPS and cell tower movements), social activity (measured by the number and duration of outgoing calls and the number of SMS sent per day)	Clinical symptoms were related to smartphone-based objective and subjective measurements. Physical activity represents a warning sign for phase transitions in BD.
[28]	Pilot study	10 pts, 18–65 yy	BD (unspecified type) (ICD- 10) willingness and ability to deal with modern smart-phones	To develop a smartphone sensor-based app that automatically record all BD-relevant data in the phone background without requiring any input by the users.	Length of study: 12 weeks Intervention group: 10 participants Type of app: Android app (name unspecified) and “openSmile” for extract voice features Type of recorded data: passive smartphone data (phone call features, speech and voice features, GPS data, acceleration, movement features)	The system developed is able to detect early changes in the state of a BD patient.
[29]	Prospective community study	49 enrolled pts, of which 36 included (27 F, 9 M)	BD unspecified type (*n =* 22) HC (*n =* 14) (DSM-IV-TR)	To identify periods of depression using geolocation movements recorded from mobile phones.	Length of study: 3 months Intervention group: 36 pts Type of app: (a) True Colours (b) custom app that records geographic location Type of recorded data: (a) weekly subjective self-assessment (b) passive smartphone geolocation data	It is possible to detect depressive episodes in BD subjects with 85% accuracy by using geographic location recordings.
[30]	14-week field trial	14 enrolled pts, of which 12 included (7 F, 5 M), 20–51yy	BD (unspecified type)	To develop a healthcare system (MONARCA system), that allows BD subjects to monitor and get feedback on their health and wellness.	Length of study: 14 weeks. Intervention group: 14 pts Type of app: MONARCA Type of recorded data: (a) self-assessment data (mood, sleep, subjective activity, medicine adherence) (b) Passive smartphone data: physical activity (measured by the accelerometer), social activity (measured by the number of in- and outgoing phone calls and text messages)	Compared to using paper-based forms, the adherence to self-assessment improved. MONARCA app was considered very easy to use, by reaching a very high perceived usefulness by patients.
[31]	RCT	18 enrolled pts (age and gender unspecified)	BD (unspecified type)	To investigate if changes in behavior of patients with BD can be captured through the analysis of smartphone usage.	Length of study: 5 months Intervention group: 18 pts Type of app: MONARCA Type of recorded data: (a) daily self-assessment; (b) passive smartphone data (number and type of running apps, times screen status is on, Amount of time patients interact with smartphone)	Strong correlation of patterns of app usage with different aspects of patients self-reported state like the mood, the sleep and the level of irritability.
[32]	RCT	123 enrolled pts, of which 78 included, 18–60 yy	BD (unspecified type) (ICD-10)	To investigate whether the use of daily electronic self-monitoring using smartphones reduces depressive and manic symptoms in BD patients.	Length of study: 6 months Intervention group: 39 pts Control group: 39 patients Type of app: MONARCA Type of recorded data: smartphone-based self-monitoring data	MONARCA system may be an effective tool able to early recognize warning signs and symptoms of a hypomanic/manic episode in BD subjects.
[33]	Prospective cohort study	66 pts	BD (unspecified type) (*n =* 29) HC (*n =* 37) (ICD-10)	To investigate whether objective smartphone data could discriminate between patients with BD and HC.	Length of study: 12 weeks Intervention group: 66 pts Type of app: MONARCA Type of recorded data: (a) daily smartphone-based self-monitoring data (b) Passive smartphone data (number of calls and text messages/day, the duration of phone calls, the number of times the smartphones’ screen was turned ‘on/off’ per day, the duration the smartphone screen was ‘on’ per day)	Automatically generated objective smartphone data (the number of text messages/day, the duration of phone calls/day) were increased in patients with BD compared with HC.
[34]	RCT	735 enrolled pts, of which 129 included	BD (unspecified type) (ICD-10)	To investigated the effect of a new smartphone-based system on the severity of depressive and manic symptoms in BD.	Length of study: 9 months Intervention group: 85 pts Control group: 44 pts Type of app: MONARCA Type of recorded data: (a) daily smartphone-based self-monitoring data (b) Passive smartphone data	Smartphone-based monitoring and real-time mood prediction, did not reduce the severity of depressive and manic symptoms. Pts in the intervention group reported improved quality of life and reduced perceived stress. Patients in the intervention group had higher risk of depressive episodes. and reduced risk of manic episodes.
[35]	Prospective cohort study	300 included pts	BD Type I (DSM-V) agreed to commence a trial of lithium treatment	To examine the early prediction of lithium response, non-response and tolerability by combining systematic clinical syndrome subtyping with examination of multi-modal biomarkers including omics, neuroimaging, and actigraphy.	Length of study: 24 months Intervention group: 300 pts Type of app: unspecified Type of recorded data: (a) Daily subjective self-rating (e.g., mood, energy, activity) (b) Continuous actigraphic sleep–wake cycles data (c) home-based measurement of salivary lithium levels (through a prototype device)	Study in progress. The project may help to refine the clinical response phenotype and could translate into the personalization of lithium treatment.

Pts: participants; BD: Bipolar Disorder; BD-I: Bipolar Disorder-type I; BD-II: Bipolar Disorder-type II; HC: Healthy Controls; n: sample size.

## References

[1] American Psychiatric Association (2013). Diagnostic and Statistical Manual of Mental Disorders (DSM-5®).

[2] Harrison P., Cipriani A., Harmer C.J., Nobre A.C., Saunders K.E.A., Goodwin G.M., Geddes J.R. (2016). Innovative approaches to bipolar disorder and its treatment. Ann. N. Y. Acad. Sci..

[3] Rowland T., Marwaha S. (2018). Epidemiology and risk factors for bipolar disorder. Ther. Adv. Psychopharmacol..

[4] Dome P., Rihmer Z., Gonda X. (2019). Dome Suicide Risk in Bipolar Disorder: A Brief Review. Medicina.

[5] Hayes J.F., Miles J., Walters K., King M., Osborn D.P.J. (2015). A systematic review and meta-analysis of premature mortality in bipolar affective disorder. Acta Psychiatr. Scand..

[6] Insel T.R. (2017). Digital phenotyping: Technology for a new science of behavior. JAMA.

[7] Onnela J.-P., Rauch S.L. (2016). Harnessing Smartphone-Based Digital Phenotyping to Enhance Behavioral and Mental Health. Neuropsychopharmacology.

[8] Brietzke E., Hawken E.R., Idzikowski M., Pong J., Kennedy S.H., Brietzke E. (2019). Integrating digital phenotyping in clinical characterization of individuals with mood disorders. Neurosci. Biobehav. Rev..

[9] Jain S.H., Powers B.W., Hawkins J.B., Brownstein J.S. (2015). The digital phenotype. Nat. Biotechnol..

[10] Lydon-Staley D.M., Barnett I., Satterthwaite T.D., Bassett D.S. (2019). Digital phenotyping for psychiatry: Accommodating data and theory with network science methodologies. Curr. Opin. Biomed. Eng..

[11] Jacobson N.C., Summers B.J., Wilhelm S. (2020). Digital Biomarkers of Social Anxiety Severity: Digital Phenotyping Using Passive Smartphone Sensors. J. Med. Internet Res..

[12] Barnett I., Torous J., Staples P., Sandoval L., Keshavan M., Onnela J.-P. (2018). Relapse prediction in schizophrenia through digital phenotyping: A pilot study. Neuropsychopharmacology.

[13] Kleiman E.M., Turner B.J., Fedor S., Beale E.E., Picard R.W., Huffman J.C., Nock M.K. (2018). Digital phenotyping of suicidal thoughts. Depress. Anxiety.

[14] Fernandes B.S., Karmakar C., Tamouza R., Tran T., Yearwood J., Hamdani N., Laouamri H., Richard J.-R., Yolken R., Berk M. (2020). Precision psychiatry with immunological and cognitive biomarkers: A multi-domain prediction for the diagnosis of bipolar disorder or schizophrenia using machine learning. Transl. Psychiatry.

[15] Ozomaro U., Wahlestedt C., Nemeroff C.B. (2013). Personalized medicine in psychiatry: Problems and promises. BMC Med..

[16] Perna G., Grassi M., Caldirola D., Nemeroff C.B. (2017). The revolution of personalized psychiatry: Will technology make it happen sooner?. Psychol. Med..

[17] Salagre E., Dodd S., Aedo A., Rosa A., Amoretti S., Pinzon J., Reinares M., Berk M., Kapczinski F.P., Vieta E. (2018). Toward Precision Psychiatry in Bipolar Disorder: Staging 2.0. Front. Psychiatry.

[18] McInnis M., Gideon J., Provost E.M. (2017). Digital Phenotyping in Bipolar Disorder. Eur. Neuropsychopharmacol..

[19] Cho C.-H., Lee T., Kim M.-G., In H.P., Kim L., Lee H.-J. (2019). Mood Prediction of Patients With Mood Disorders by Machine Learning Using Passive Digital Phenotypes Based on the Circadian Rhythm: Prospective Observational Cohort Study. J. Med. Internet Res..

[20] Zulueta J., Piscitello A., Rasic M., Easter R., Babu P., Langenecker S.A., McInnis M.G., Ajilore O., Nelson P.C., Ryan K.A. (2018). Predicting Mood Disturbance Severity with Mobile Phone Keystroke Metadata: A BiAffect Digital Phenotyping Study. J. Med. Internet Res..

[21] Hidalgo-Mazzei D., Hassani H., Ostacher M., Graham A., Busk J., Faurholt-Jepsen M., Frost M., Bardram J.E., Kessing L.V., Winther O. (2020). Forecasting Mood in Bipolar Disorder From Smartphone Self-assessments: Hierarchical Bayesian Approach. JMIR mHealth uHealth.

[22] Dargél A.A., Mosconi E., Masson M., Plaze M., Taieb F., Von Platen C., Buivan T.P., Henry C. (2020). Toi Même: A mHealth Platform for Measuring Bipolar Illness Activity-Feasibility Study Protocol. JMIR Res. Protoc..

[23] Higgins J.P., Green S. (2011). Cochrane Handbook for Systematic Reviews of Interventions.

[24] Liberati A., Altman D.G., Tetzlaff J., Mulrow C., Gøtzsche P.C., Ioannidis J.P.A., Clarke M., Devereaux P.J., Kleijnen J., Moher D. (2009). The PRISMA statement for reporting systematic reviews and meta-analyses of studies that evaluate healthcare interventions: Explanation and elaboration. BMJ.

[25] Cao B., Zheng L., Zhang C., Yu P.S., Piscitello A., Zulueta J., Leow A.D. Deepmood: Modeling mobile phone typing dynamics for mood detection. Proceedings of the 23rd ACM SIGKDD International Conference on Knowledge Discovery and Data Mining.

[26] Gideon J., Provost E.M., McInnis M. (2016). Mood state prediction from speech of varying acoustic quality for individuals with bipolar disorder. Proceedings of the 2016 IEEE International Conference on Acoustics, Speech and Signal Processing (ICASSP).

[27] Karam Z.N., Provost E.M., Singh S., Montgomery J., Archer C., Harrington G., McInnis M.G. (2014). Ecologically valid long-term mood monitoring of individuals with bipolar disorder using speech. Proceedings of the 2014 IEEE International Conference on Acoustics, Speech and Signal Processing (ICASSP).

[28] Muaremi A., Gravenhorst F., Grünerbl A., Arnrich B., Tröster G. (2014). Assessing Bipolar Episodes Using Speech Cues Derived from Phone Calls. Proceedings of the International Symposium on Pervasive Computing Paradigms for Mental Health.

[29] Abdullah S., Matthews M., Frank E., Doherty G., Gay G., Choudhury T. (2016). Automatic detection of social rhythms in bipolar disorder. J. Am. Med. Inform. Assoc..

[30] Beiwinkel T., Kindermann S., Maier A., Kerl C., Moock J., Barbian G., Rössler W., Faurholt-Jepsen M., Mayora O., Buntrock C. (2016). Using Smartphones to Monitor Bipolar Disorder Symptoms: A Pilot Study. JMIR Ment. Health.

[31] Grunerbl A., Muaremi A., Osmani V., Bahle G., Ohler S., Troster G., Mayora O., Haring C., Lukowicz P. (2014). Smartphone-Based Recognition of States and State Changes in Bipolar Disorder Patients. IEEE J. Biomed. Health Inform..

[32] Palmius N., Tsanas A., Saunders K.E.A., Bilderbeck A.C., Geddes J.R., Goodwin G.M., De Vos M. (2016). Detecting Bipolar Depression From Geographic Location Data. IEEE Trans. Biomed. Eng..

[33] Bardram J.E., Frost M., Szántó K., Faurholt-Jepsen M., Vinberg M., Kessing L.V. Designing mobile health technology for bipolar disorder. Proceedings of the SIGCHI Conference on Human Factors in Computing Systems.

[34] Alvarez-Lozano J., Osmani V., Mayora O., Frost M., Bardram J., Faurholt-Jepsen M., Kessing L.V. (2014). Tell me your apps and I will tell you your mood. Proceedings of the 7th International Conference on PErvasive Technologies Related to Assistive Environments.

[35] Faurholt-Jepsen M., Frost M., Ritz C., Christensen E.M., Jacoby A.S., Mikkelsen R.L., Knorr U., Bardram J.E., Vinberg M., Kessing L.V. (2015). Daily electronic self-monitoring in bipolar disorder using smartphones—The MONARCA I trial: A randomized, placebo-controlled, single-blind, parallel group trial. Psychol. Med..

[36] Faurholt-Jepsen M., Busk J., Þórarinsdóttir H., Frost M., Bardram J.E., Vinberg M., Kessing L.V. (2018). Objective smartphone data as a potential diagnostic marker of bipolar disorder. Aust. N. Zeal. J. Psychiatry.

[37] Faurholt-Jepsen M., Frost M., Christensen E.M., Bardram J.E., Vinberg M., Kessing L.V. (2019). The effect of smartphone-based monitoring on illness activity in bipolar disorder: The MONARCA II randomized controlled single-blinded trial. Psychol. Med..

[38] Scott J., Hidalgo-Mazzei D., Strawbridge R., Young A.H., Resche-Rigon M., Etain B., Andreassen O.A., Bauer M., Bennabi D., Blamire A.M. (2019). Prospective cohort study of early biosignatures of response to lithium in bipolar-I-disorders: Overview of the H2020-funded R-LiNK initiative. Int. J. Bipolar Disord..

[39] Cho C.-H., Ahn Y.-M., Kim S.J., Ha T.H., Jeon H.J., Cha B., Moon E., Park D.Y., Baek J.H., Kang H.-J. (2016). Design and Methods of the Mood Disorder Cohort Research Consortium (MDCRC) Study. Psychiatry Investig..

[40] Faurholt-Jepsen M., Vinberg M., Frost M., Christensen E.M., Bardram J.E., Kessing L.V. (2014). Daily electronic monitoring of subjective and objective measures of illness activity in bipolar disorder using smartphones—The MONARCA II trial protocol: A randomized controlled single-blind parallel-group trial. BMC Psychiatry.

[41] Assoc A.P. (1988). Diagnostic and Statistical Manual of Mental Disorders. Alzheimer Dis. Assoc. Disord..

[42] McInnis M., Assari S., Kamali M., Ryan K., Langenecker S.A., Saunders E.F.H., Versha K., Evans S.J., O’Shea K.S., Provost E.M. (2017). Cohort Profile: The Heinz C. Prechter Longitudinal Study of Bipolar Disorder. Int. J. Epidemiol..

[43] Langenecker S.A., Saunders E.F., Kade A.M., Ransom M.T., McInnis M.G. (2010). Intermediate: Cognitive phenotypes in bipolar disorder. J. Affect. Disord..

[44] Rush A., Trivedi M.H., Ibrahim H.M., Carmody T.J., Arnow B., Klein D.N., Markowitz J.C., Ninan P.T., Kornstein S., Manber R. (2003). The 16-Item quick inventory of depressive symptomatology (QIDS), clinician rating (QIDS-C), and self-report (QIDS-SR): A psychometric evaluation in patients with chronic major depression. Biol. Psychiatry.

[45] Huckvale K., Venkatesh S., Christensen H. (2019). Toward clinical digital phenotyping: A timely opportunity to consider purpose, quality, and safety. Npj Digit. Med..

[46] Pew Research (2018). Mobile Fact Sheet. http://www.pewinternet.org/fact-sheet/mobile/.

[47] Torous J., Kiang M.V., Lorme J., Onnela J.-P. (2016). New Tools for New Research in Psychiatry: A Scalable and Customizable Platform to Empower Data Driven Smartphone Research. JMIR Ment. Health.

[48] Smith D.G. (2018). Digital phenotyping approaches and mobile devices enhance CNS biopharmaceutical research and development. Neuropsychopharmacology.

[49] Marwaha S., Price C., Scott J., Weich S., Cairns A., Dale J., Winsper C., Broome M.R. (2018). Affective instability in those with and without mental disorders: A case control study. J. Affect. Disord..

[50] Selby E.A., Yen S., Spirito A. (2012). Time varying prediction of thoughts of death and suicidal ideation in adolescents: Weekly ratings over 6-month follow-up. J. Clin. Child Adolesc. Psychol..

[51] Bauer M., Glenn T., Monteith S., Bauer R., Whybrow P.C., Geddes J.R. (2017). Ethical perspectives on recommending digital technology for patients with mental illness. Int. J. Bipolar Disord..

[52] Insel T.R. (2014). The NIMH Research Domain Criteria (RDoC) Project: Precision Medicine for Psychiatry. Am. J. Psychiatry.

[53] Insel T.R. (2018). Digital phenotyping: A global tool for psychiatry. World Psychiatry.

[54] Torous J., Onnela J.-P., Keshavan M. (2017). New dimensions and new tools to realize the potential of RDoC: Digital phenotyping via smartphones and connected devices. Transl. Psychiatry.

[55] Nicholas J., Larsen M.E., Proudfoot J., Christensen H. (2015). Mobile Apps for Bipolar Disorder: A Systematic Review of Features and Content Quality. J. Med. Internet Res..

[56] Klugman C.M., Dunn L.B., Schwartz J., Cohen I.G. (2018). The Ethics of Smart Pills and Self-Acting Devices: Autonomy, Truth-Telling, and Trust at the Dawn of Digital Medicine. Am. J. Bioeth..

